# Value of tissue Doppler-derived Tei index and two-dimensional speckle tracking imaging derived longitudinal strain on predicting outcome of patients with light-chain cardiac amyloidosis

**DOI:** 10.1007/s10554-017-1075-5

**Published:** 2017-03-06

**Authors:** Dan Liu, Kai Hu, Sebastian Herrmann, Maja Cikes, Georg Ertl, Frank Weidemann, Stefan Störk, Peter Nordbeck

**Affiliations:** 1 Comprehensive Heart Failure Center, University of Würzburg, Würzburg, Germany; 2 Department of Internal Medicine I, University Hospital Würzburg, Oberdürrbacher Str. 6, 97080 Würzburg, Germany; 3 Department for Cardiovascular Diseases, University Hospital Center Zagreb and School of Medicine, University of Zagreb, Zagreb, Croatia; 4 Medical Clinic II, Katharinen-Hospital Unna, Unna, Germany

**Keywords:** Tei index, Strain rate, Cardiomyopathy, Tissue Doppler echocardiography

## Abstract

Prognosis of patients with light-chain cardiac amyloidosis (AL-CA) is poor. Speckle tracking imaging (STI) derived longitudinal deformation parameters and Doppler-derived left ventricular (LV) Tei index are valuable predictors of outcome in patients with AL-CA. We estimated the prognostic utility of Tei index and deformation parameters in 58 comprehensively phenotyped patients with AL-CA after a median follow-up of 365 days (quartiles 121, 365 days). The primary end point was all-cause mortality. 19 (33%) patients died during follow-up. Tei index (0.89 ± 0.29 vs. 0.61 ± 0.16, p < 0.001) and E to global early diastolic strain rate ratio (E/GLSR_dias_) were higher while global longitudinal systolic strain (GLS_sys_) was lower in non-survivors than in survivors (all p < 0.05). Tei index, NYHA functional class, GLS_sys_ and E/GLSR_dias_ were independent predictors of all-cause mortality risk, and Tei index ≥0.9 (HR 7.01, 95% CI 2.43–20.21, p < 0.001) was the best predictor of poor outcome. Combining Tei index and GLS_sys_ yielded the best results on predicting death within 1 year (100% with Tei index ≥0.9 and GLS_sys_ ≤13%) or survival (95% with Tei index ≤0.9 and GLS_sys_ ≥13%). We conclude that 1-year mortality risk in AL-CA patients can be reliably predicted using Tei index or deformation parameters, with combined analysis offering best performance.

## Introduction

Primary amyloidosis formed from immunoglobulin light chain (AL) is one of the main types of systemic amyloidosis, most commonly involving the kidneys and the heart [1]. Cardiac involvement (CA) is a leading cause of morbidity and mortality in AL amyloidosis [2].

Echocardiography plays an important role in the diagnosis and disease progression monitoring of CA patients. Typical morphological findings of CA include left ventricular (LV) hypertrophy, biatrial enlargement, and small pericardial effusion. Diastolic dysfunction usually occurs at the early stage, whereas LV ejection fraction remains normal until the late stage. Previous studies have reported the diagnostic and prognostic utility of conventional echocardiographic parameters and advanced deformation parameters in patients with AL-CA [3–5]. Results from our group and others demonstrated that longitudinal systolic and diastolic deformation parameters derived from speckle tracking imaging (STI) could effectively predict the outcome of AL-CA patients [6–8].

As early as 1996, Tei and colleagues reported that the pulsed Doppler-derived Tei index, a simple parameter reflecting both systolic and diastolic myocardial performance, correlated with global cardiac dysfunction and was a valuable outcome predictor in CA patients [9, 10]. Tei index can be calculated by both pulsed Doppler and tissue Doppler (TD) methods. The major limitation of the Tei index assessment using pulsed Doppler is that it cannot be measured within one cardiac cycle, while the TD method enables measurement of both relaxation and contraction velocities simultaneously [11].

In this study, we compared the prognostic utility between TD derived Tei index and STI derived deformation parameters as well as the incremental impact of these parameters in patients with AL-CA during 1-year of follow-up.

## Methods

### Study population and clinical follow-up

A total of 58 consecutive biopsy-proven AL-CA patients referred to the University Hospital Würzburg and University Hospital Center Zagreb between January 2005 and October 2014 were enrolled in this retrospective study. Cardiac involvement was diagnosed according to echocardiographic findings as described in our previous studies [6, 12]. Patients with coronary artery disease, higher grade cardiac valve diseases, and other cardiac pathologies were excluded. Written informed consent was obtained from all patients or their guardians. The study was approved by the local Ethics Committee at the University of Würzburg and University Hospital Center Zagreb, and conducted in accordance to the Declaration of Helsinki. All patients completed a 1-year follow-up performed by clinical visit or telephone interview. The primary end point was all-cause mortality.

### Standard echocardiographic measurements

A standard echocardiographic examination was performed in all patients (GE Vingmed Vivid 7 or Vivid 9, Horten, Norway). Measurements were performed off-line using a remote workstation (EchoPAC version 112, GE, Horten, Norway). LV end-diastolic dimension (LVEDD), end-diastolic thickness of the posterior wall (LVPWd) and the septum (IVSd), LV stroke volume, and fractional shortening (FS) were measured using M-mode in the parasternal LV long axis view. Left atrial end-systolic diameter was measured in the parasternal long-axis view. LV biplane Simpson method ejection fraction (EF) was measured in apical 4- and 2-chamber views. Septal mitral annular plane systolic excursion (MAPSE) was measured by M-mode in apical 4-chamber view. LV filling pattern was assessed according to current guidelines [13]. Pulsed Doppler was performed in the apical 4-chamber view to obtain mitral inflow velocities. Peak velocity of early (E) and atrial (A) diastolic filling and deceleration time of E wave (DT) were measured and the E/A ratio calculated. Tissue Doppler early diastolic mitral annular velocity (E′) was acquired at the septal mitral annulus.

### Tissue Doppler derived Tei index measurement

**Fig. 1:**
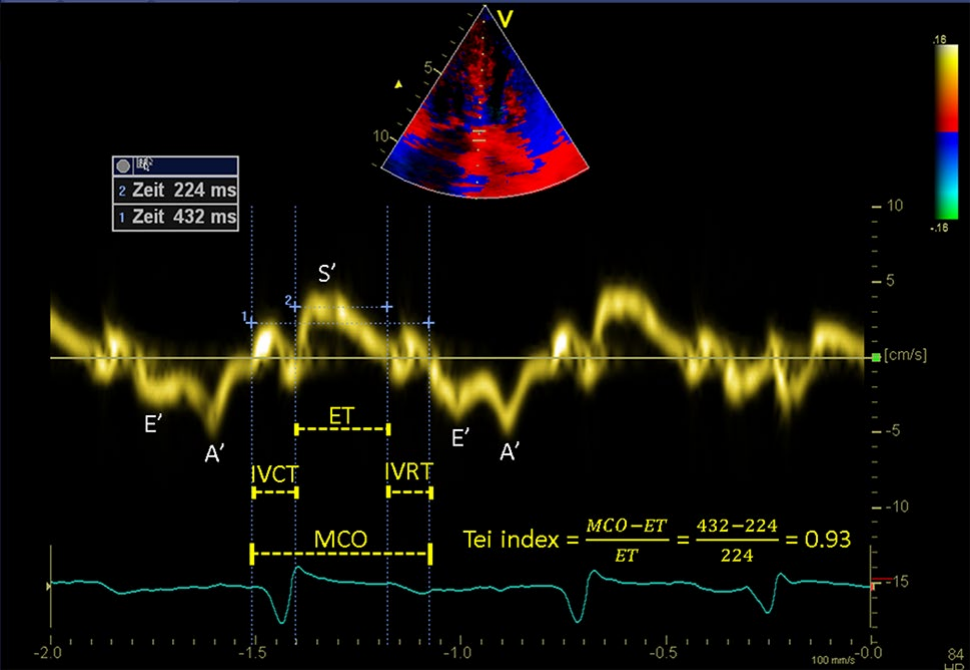
Example of a Tissue Doppler derived Tei index. Time intervals of left ventricle are measured with tissue Doppler imaging and the Tei index is calculated by the formula: (MCO-ET)/ET. *MCO* mitral valve closure-to-opening time, *ET* ejection time, *IVCT* isovolumetric contraction time, *IVRT* isovolumetric relaxation time, *S*′ peak systolic septal mitral annular velocity, *E*′ peak early-diastolic septal mitral annular velocity, *A*′ peak late-diastolic septal mitral annular velocity

Interval measurements were performed within one cardiac cycle as illustrated in Fig. 1. The TD derived Tei index was calculated: (MCO-ET)/ET, where MCO is the time interval from the end of the TD late diastolic annular velocity A′ wave to the onset of the E′ wave, representing the mitral valve closure-to-opening time, and ET is the time from the onset to the end of the TD systolic annular velocity S′ wave, reflecting the ejection time of the LV. Isovolumetric contraction time (IVCT) was measured from the end of the A′ wave to the onset of the S′ wave, and isovolumetric relaxation time (IVRT) was measured from the end of the S′ wave to the onset of the E′ wave. The Tei index values from 3 (sinus rhythm) to 5 (atrial fibrillation) cardiac cycles were averaged.

### STI-derived systolic and diastolic deformation

Standard 2D grey scale apical 4-chamber view was recorded and STI was analyzed off-line using EchoPAC post processing program version 112 (GE, Horten, Norway) as previously described [7]. Global longitudinal systolic peak strain rate (GLSR_sys_) and strain (GLS_sys_) at the midline, and global longitudinal peak early diastolic strain rate (GLSR_dias_) of 6 segments were measured in the apical 4-chamber view (including the basal, mid and apical segments of the septal and lateral walls). The E/GLSR_dias_ ratio was calculated as the pulsed Doppler detected E velocity divided by the GLSR_dias_. The ratio between apical and basal LS_sys_ (LSsys_api/bas_) of the septum was calculated as septal apical LS_sys_ divided by septal basal LS_sys_.

### Statistical analysis

Continuous variables were expressed as mean ± standard deviation (SD) or median (quartiles). Categorical variables were presented as percentages. Non-normally distributed variables, including N-terminal prohormone brain natriuretic peptide (NT-proBNP), E/GLSR_dias_ ratio, and LSsys_api/bas_, were normalized prior to analysis. Differences on continuous data between groups were compared using Student’s *t* test. Categorical data were compared using Chi square test.

Survival curves were plotted by the Kaplan–Meier method, and compared by log-rank tests. Cox proportional hazards regression was used in multivariable analyses, and hazard ratios (HR) with 95% confidence intervals (CI) were reported. Outcome was adjusted for age, gender, and body mass index. The performance of different prediction models was judged on the basis of the c-statistic (area under the receiver operating characteristic curves). Statistical significance was defined as p < 0.05. Statistical analysis was performed using IBM SPSS, version 23 for Windows (IBM Corp., New York, USA).

## Results

### Clinical parameters

Fifty-eight AL-CA patients were included (mean age 64 ± 10 years, 53% male). Their clinical data are listed in Table 1. The median duration of follow-up was 365 (quartiles: 121, 365) days. During follow-up, 19 CA patients (33%) died. Percent of NYHA functional class III–IV was significantly higher in non-survivors than survivors (84% vs 36%, p = 0.001). Serum estimated glomerular filtration rate (eGFR) was similar between both groups. Serum NT-proBNP levels were markedly elevated in the whole cohort (median 4543 pg/ml) and tended to be higher in non-survivors compared with survivors (8062 vs. 2595 pg/ml, p = 0.181).

**Table 1:** Clinical characteristics

	Total	Survivors	Non-survivors	P value
	N = 58	N = 39	N = 19	
Age (years)	64 ± 10	65 ± 9	64 ± 11	0.618
Male (n, %)	31, 53%	23, 59%	8, 42%	0.227
BMI (kg/m²)	25 ± 4	24 ± 4	26 ± 5	0.123
Systolic blood pressure (mmHg)	116 ± 20	118 ± 18	112 ± 24	0.352
Diastolic blood pressure (mmHg)	73 ± 13	74 ± 12	70 ± 13	0.305
Heart rate (beats/min)	79 ± 12	77 ± 11	84 ± 13	0.070
NYHA functional class (n, %)				0.019
I	2, 3%	1, 3%	1, 5%	
II	26, 45%	24, 61%	2, 11%	
III	24, 42%	12, 31%	12, 63%	
IV	6, 10%	2, 5%	4, 21%	
NYHA class III–IV (n, %)	30, 52%	14, 36%	16, 84%	0.001
eGFR (ml/min/1.73 m²)	62 ± 30	64 ± 32	59 ± 26	0.526
Albumin (g/dl)	3.6 ± 0.8	3.6 ± 0.8	3.8 ± 0.6	0.169
NT-proBNP (pg/ml)	4543 (1615–13,505)	2595 (955–8765)	8062 (3533–16,451)	0.181

*BMI* body mass index, *NYHA* New York Heart Association, *eGFR* estimated glomerular filtration rate, *NT-proBNP* N-terminal of the prohormone brain natriuretic peptide

### Echocardiographic parameters

Conventional echocardiographic data were listed in Table 2. LV-FS and LV-EF remained normal or slightly reduced, but were significantly lower in non-survivors than survivors. Septal MAPSE was significantly reduced in the whole cohort as compared to normal value (≥10 mm) [14], and was significantly lower in non-survivors than survivors (p = 0.005). The prevalence of advanced diastolic dysfunction (i.e., pseudonormal or restrictive diastolic filling pattern) was also significantly higher in non-survivors than in survivors (87% vs. 54%, p = 0.031).

**Table 2:** Echocardiographic characteristics

	Total	Survivors	Non-survivors	P value
	N = 58	N = 39	N = 19	
Standard measurements				
LV-EDD (mm)	45 ± 7	45 ± 7	45 ± 8	0.789
IVSd (mm)	14.7 ± 3.1	14.1 ± 3.1	15.6 ± 2.9	0.082
LV-PWd (mm)	14.3 ± 3.2	13.6 ± 3.2	15.1 ± 2.5	0.083
LV-FS (%)	27 ± 9	30 ± 7	22 ± 8	<0.001
LV-EF (%)	54 ± 12	58 ± 10	47 ± 13	0.001
SV (ml)	41 ± 16	42 ± 16	40 ± 17	0.569
Septal MAPSE (mm)	5.9 ± 2.6	6.6 ± 2.5	4.6 ± 2.2	0.005
LA (mm)	43 ± 7	43 ± 8	44 ± 6	0.487
E (cm/s)	0.89 ± 0.23	0.88 ± 0.25	0.92 ± 0.21	0.479
E/A ratio	1.68 ± 0.91	1.56 ± 0.98	1.84 ± 0.73	0.334
DT (ms)	161 ± 57	171 ± 58	136 ± 42	0.031
E/E′ ratio	23.2 ± 10.3	21.7 ± 10.5	27.6 ± 9.6	0.074
Diastolic filling pattern (n, %)				0.170
Normal	1, 2%	1, 3%	0, 0%	
Abnormal relaxation	16, 28%	14, 36%	2, 10%	
Pseudonormal	16, 28%	10, 26%	6, 32%	
Restrictive	15, 26%	8, 20%	7, 37%	
Atrial fibrillation	10, 17%	6, 15%	4, 21%	
Tissue Doppler derived-Tei index				
MCO (ms)	414 ± 59	412 ± 49	418 ± 76	0.712
ET (ms)	246 ± 35	257 ± 29	223 ± 34	<0.001
Tei index	0.70 ± 0.25	0.61 ± 0.16	0.89 ± 0.29	0.001
IVCT (ms)	79 ± 33	70 ± 20	96 ± 44	0.026
IVRT (ms)	92 ± 27	88 ± 24	98 ± 31	0.185
Longitudinal systolic and diastolic deformation				
GLS_sys_ (%)	−10.7 ± 4.3	−12.1 ± 3.6	−8.3 ± 4.6	0.001
GLSR_sys_ (S^− 1^)	−0.72 ± 0.29	−0.80 ± 0.26	−0.60 ± 0.32	0.013
Septal LSsys_api/bas_	3.09 (2.10–4.64)	2.64 (1.99–4.03)	3.36 (2.90–5.68)	0.062
GLSR_dias_ (S^− 1^)	0.79 ± 0.35	0.87 ± 0.30	0.67 ± 0.40	0.039
E/GLSR_dias_	1.21 (0.84–1.59)	1.00 (0.76–1.39)	1.65 (0.96–2.44)	0.004

*LV-EDD* left ventricular end-diastolic diameter, *IVSd* end-diastolic interventricular septal wall thickness, *LV-PWd* end-diastolic left ventricular posterior wall thickness, *LV-FS* fractional shortening, *LV-EF* ejection fraction, *SV* stroke volume, *MAPSE* mitral annular plane systolic excursion, *LA* left atrial, *E* peak early diastolic mitral inflow velocity, *E*/*A* peak early to late diastolic mitral inflow velocity ratio, DT deceleration time of E wave, *E*/*E*′ peak early diastolic mitral inflow velocity to tissue Doppler mitral annular velocity ratio, *MCO* mitral valve closure-to-opening time, *ET* ejection time, Tei index (MCO-ET)/ET, *IVCT* isovolumetric contract time, *IVRT* isovolumetric relaxation time, *GLS*_*sys*_ global longitudinal systolic strain, *GLSR*_*sys*_ global longitudinal systolic strain rate, *LSsys*_*api*/bas_ The ratio between apical and basal *LS*_*sys*_ of the septum, *GLSR*_*dias*_ global early diastolic strain rate, E/*GLSR*_*dias*_ peak early diastolic mitral inflow velocity to global early diastolic strain rate ratio

### Tissue Doppler derived Tei index

TD derived-Tei index was 0.70 ± 0.25 in the whole cohort, which is markedly higher than normal reference value (<0.40) [10]. Tei index was significantly higher in non-survivors than in survivors (0.89 ± 0.29 vs. 0.61 ± 0.16, p = 0.001).

### STI derived systolic and diastolic deformation parameters

As shown in Table 2, GLSR_sys_ and GLS_sys_ as well as GLSR_dias_ were significantly lower in non-survivors than in survivors (all p < 0.05). The E/GLSR_dias_ ratio was significantly higher in non-survivors than in survivors (p < 0.05).

### Survival analysis

**Fig. 2:**
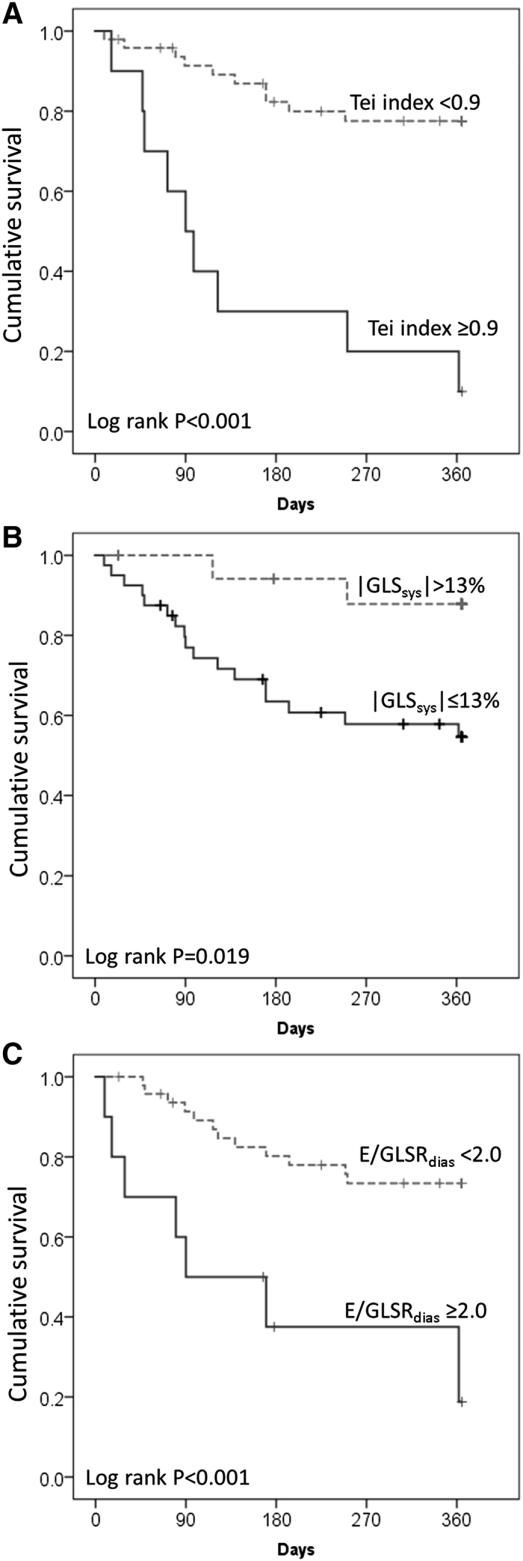
Kaplan–Meier estimation of Tei index, GLS_sys_, and E/GLSR_dias_ for predicting all-cause mortality in patients with light-chain cardiac amyloidosis (AL-CA). Note that increased Tei index (**a** ≥0.9, P < 0.001), reduced GLS_sys_ (**b** ≤13%, p = 0.019), or increased E/GLSR_dias_ ratio (**c** ≥2.0, P < 0.001) was associated with significantly increased risk of all-cause death within 1 year in patients with AL-CA. *GLS*_*sys*_ global longitudinal systolic strain, E/*GLSR*_*dias*_ peak early diastolic mitral inflow velocity to global early diastolic strain rate ratio

Survival analysis (Table 3) showed that NYHA class was an independent clinical prognostic marker in this cohort. Among conventional echocardiographic and deformation parameters, after adjustment for age, gender and body mass index, each of the following markers predicted an increased 1-year mortality risk: LV-FS, LV-EF, septal MAPSE, DT, E/E′, ET, IVCT, Tei index, GLS_sys_ and GLSR_sys_, as well as E/GLSR_sys_ ratio. The optimal cut-off value for Tei index was 0.9 with a specificity of 100% for predicting 1-year mortality. Increased Tei index (≥0.9 vs. <0.9, log rank p < 0.001), increased E/GLSR_dias_ ratio (≥2.0 vs. <2.0, p < 0.001), or reduced GLS_sys_ (≤13% vs. >13%, p = 0.019) in patients with AL-CA was associated with significantly increased risk of all-cause death within 1 year (Fig. 2a–c). The multivariable regression analysis including NYHA class, LV-EF, septal MAPSE, E/E′ and Tei index showed that NYHA class III–IV (HR 6.79, p = 0.025) and Tei index ≥0.9 (HR 12.33, p = 0.001) remained independent predictors in this model. Furthermore, the model including deformation predictors (GLS_sys_ and E/GLSR_dias_) and Tei index demonstrated that Tei index ≥0.9 was the best independent predictor of all-cause mortality risk (HR 7.01, p < 0.001).

**Table 3:** Cox proportional hazard regression for 1-year all-cause mortality in patients with light-chain cardiac amyloidosis

	Hazard ratio (95% confidence interval), adjusted for age, gender and BMI	P value
NYHA class III–IV vs. I–II	6.55 (1.89–22.75)	0.003
LV-FS (%)	0.91 (0.86–0.97)	0.002
LV-EF (%)	0.95 (0.91–0.99)	0.009
Septal MAPSE (mm)	0.73 (0.58–0.91)	0.006
DT (ms)	0.98 (0.97–0.99)	0.002
E/E′	1.05 (1.00–1.10)	0.038
ET (ms)	0.98 (0.96–0.99)	0.001
IVCT (ms)	1.01 (1.01–1.02)	0.002
Tei index	47.04 (7.79–284.10)	<0.001
Tei index ≥0.9 vs. <0.9	8.48 (3.27–21.99)	<0.001
GLS_sys_ (%)	1.18 (1.06–1.33)	0.004
GLSR_sys_ (S^− 1^)	8.33 (1.55–44.78)	0.014
Septal LSsys_api/bas_	1.36 (0.83–2.23)	0.223
GLSR_dias_ (S^− 1^)	0.24 (0.06–1.01)	0.051
E/GLSR_dias_	1.92 (1.19–3.08)	0.007
Model including Tei index, NYHA class and other conventional echocardiographic variables		
NYHA class III–IV vs. I–II	6.79 (1.27–36.39)	0.025
LV-EF (%)	1.04 (0.96–1.13)	0.309
Septal MAPSE (mm)	0.85 (0.68–1.06)	0.153
E/E′	0.99 (0.92–1.06)	0.788
Tei index ≥0.9 vs. <0.9	12.33 (2.92–52.04)	0.001
Model including Tei index and deformation variables		
GLS_sys_ ≤13% vs. >13%	3.88 (0.84–17.93)	0.082
E/GLSR_dias_ ≥2.0 vs. <2.0	1.44 (0.48–4.35)	0.517
Tei index ≥0.9 vs. <0.9	7.01 (2.43–20.21)	<0.001

Abbreviations as in Tables 1 and 2

Prediction model performance statistics (Table 4) revealed that Tei index alone already yielded very good predictive utility (c-statistic: 0.85). Adding deformation predictors (GLS_sys_ or E/GLSR_dias_) only slightly increased this value (c-statistic: 0.87). Other approaches yielded no further favorable utility, e.g., GLS_sys_ (c-statistic 0.77), E/GLSR_dias_ (c-statistic 0.72) or GLS_sys_ + E/GLSR_dias_ (c-statistic 0.77).

**Table 4:** Model performance statistics for Tei index and deformation predictors related 1-year all-cause mortality in patients with light-chain cardiac amyloidosis

	Overall performance	Discrimination (ROC, c-statistic)	
	Nagelkerke R²	c-statistic (95% CI)	P value
Tei index	0.40	0.85 (0.74–0.95)	<0.001
GLS_sys_ (%)	0.29	0.77 (0.64–0.90)	0.001
E/LSR_dias_	0.22	0.72 (0.58–0.87)	0.007
Tei index + GLS_sys_ (%)	0.45	0.87 (0.76–0.97)	<0.001
Tei index + E/GLSR_dias_	0.45	0.87 (0.77–0.97)	<0.001
GLS_sys_ (%) + E/GLSR_dias_	0.32	0.77 (0.63–0.90)	0.001

Abbreviations as in Table 2

**Fig. 3:**
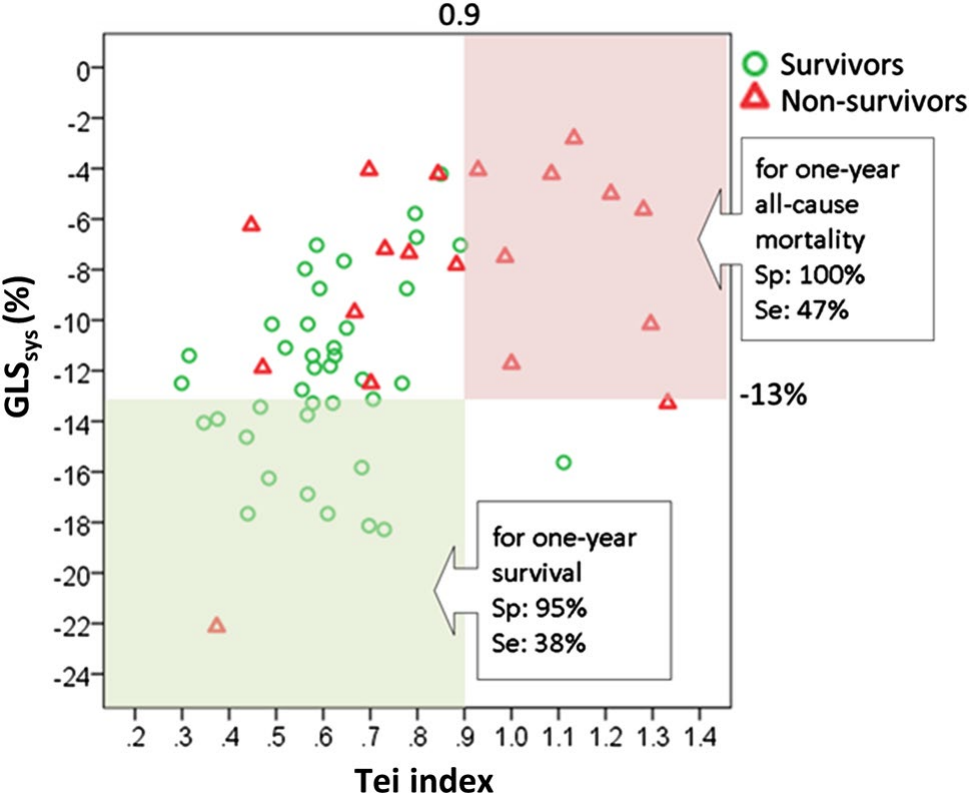
Comparison of prognostic performance of Tei index ≥0.9 and GLS_sys_ ≤13% in patients with light-chain cardiac amyloidosis. Scatter *plots* depicting the correct and wrong attribution of having died after 1 year of follow-up using certain thresholds derived from study data. Excellent prognostic performance for predicting 1-year all-cause mortality is observed using Tei index ≥0.9 plus GLS_sys_ ≤13% with a specificity of 100% and a sensitivity of 47%. *GLS*_*sys*_ global longitudinal systolic strain, *Sp* specificity, *Se* sensitivity

As shown in Fig. 3, 9 out of 9 (100%) patients with Tei index ≥0.9 and GLS_sys_ absolute value ≤13% died during 1-year follow up (specificity 100%, positive predictive value 100%; sensitivity 47%). In contrast, only 1 out of 16 (6%) patients with Tei index <0.9 and GLS_sys_ >13% died within 1 year.

**Fig. 4:**
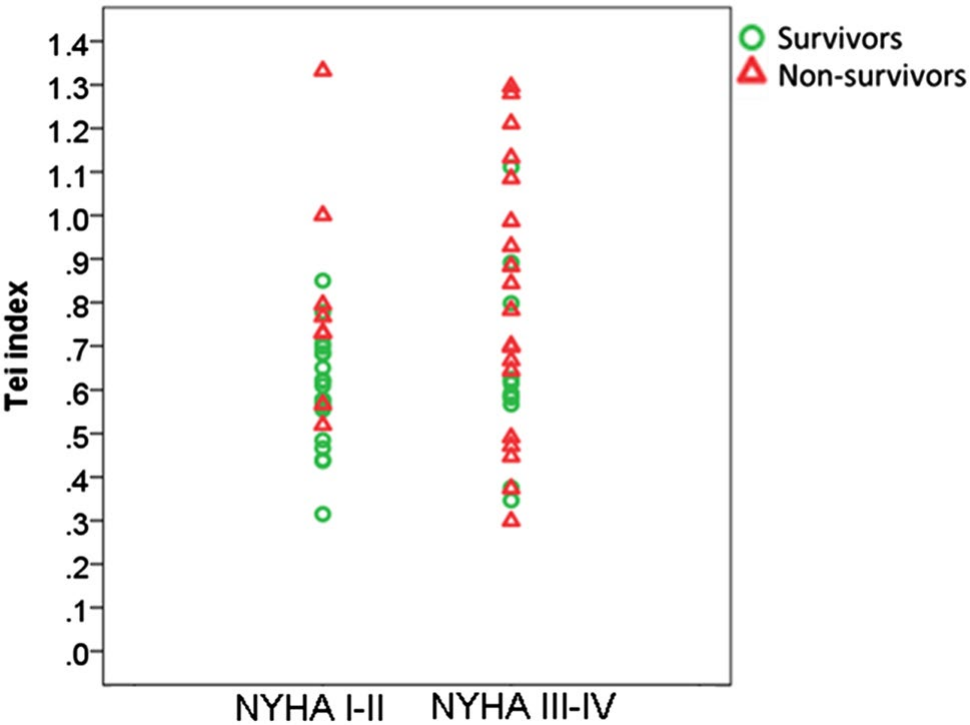
Comparison of prognostic performance of Tei index between light-chain cardiac amyloidosis patients with NYHA class I–II and III–IV. Note that in patients with NYHA class I–II, a significantly increased Tei index (≥0.9) was present in none (0/20) of survivors, but in 25% (2/8) of non-survivors (p = 0.074). Conversely, in patients with NYHA class III–IV, a normal Tei index (<0.5) was found in 18.2% (2/11) of survivors and in 26.3% (5/19) of non-survivors (p > 0.05). *NYHA class* New York Heart Association functional class

As shown in Fig. 4, in patients with NYHA class I–II, a significantly increased Tei index (≥0.9) was presented in none (0/20) of survivors, whereas in 25% (2/8) of non-survivors (p = 0.074). Conversely, in patients with NYHA class III–IV, a normal Tei index (<0.5) was found in 18.2% (2/11) of survivors and in 26.3% (5/19) of non-survivors (p > 0.05).

## Discussion

The major findings of the present study are as follows: (1) increased Tei index (≥ 0.9) served as a valuable independent predictor of all-cause mortality during 1-year follow up in AL-CA patients, which was superior to LV-EF and other conventionally derived diastolic indices, and similar to both global longitudinal systolic strain and E to global early diastolic strain rate ratio; and (2) combining Tei index (threshold ≥0.9) and GLS_sys_ (≤13%) yielded excellent prognostic utility allowing improved mortality risk stratification in AL-CA patients.

### Tei index as the best echocardiographic predictor for outcome assessment in AL-CA patients

The Tei index is an easy-to-obtain parameter, it not only reflects combined systolic and diastolic myocardial function, but is also independent of heart rate and blood pressure [10]. Although conventional measurements of systolic function such as LV-EF, LV-FS, or MAPSE proved to be feasible for prediction of outcome in this cohort, their prognostic power was weaker than Tei index. This might be due to the fact that the Tei index has the unique merit that it does not depend on ventricular geometry and image quality [15].

The potential of the Tei index as a clinically meaningful diagnostic and prognostic marker has been reported in various reports investigating symptomatic heart failure, acute myocardial infarction, dilated cardiomyopathy, and primary pulmonary hypertension [16–19]. Tei et al. also demonstrated the prognostic significance of the Tei index in patients with CA comprising patients with primary, familial and senile amyloidosis [9]. Our results extend their findings, documenting that amongst a large variety of conventional echocardiographic markers the Tei index performed best in independently predicting 1-year mortality in AL-CA patients, superior to evaluation of NYHA class, LV-EF (=global systolic function), septal MAPSE (=longitudinal global systolic function), DT or E/E′ (=diastolic function).

As stated above, the Tei index is computed by the formula (IVCT + IVRT)/ET. Tei et al. reported that the IVRT and IVCT components were significantly prolonged and the ET was significantly shortened in CA patients as compared with normal controls [9]. Consistently, the present study also demonstrated a remarkably shorter ET and longer IVCT and IVRT in non-survivors than in survivors. A possible reason for the observed ET shortening might be associated with lower end-diastolic volumes. In AL-CA, the end-diastolic volume is limited due to amyloid deposition, which consequently results in a stiff ventricle. Migrino et al. showed that ET was the most important predictor of adverse outcome in AL amyloidosis [20]. In our cohort, ET was confirmed as an independent predictor of 1-year all-cause mortality risk, and ET ≤230 ms showed a similarly good prediction performance, with a sensitivity of 68% and a specificity of 85%. Prolongation of IVCT and IVRT are also evidenced, especially in non-survivors in the present study, which indicates reduced myocardial contractility and abnormal myocardial relaxation in these patients [21]. Accordingly, the prevalence of advanced diastolic dysfunction was remarkably higher in non-survivors (87%) than survivors (54%, p < 0.05).

### Prognostic utility of Tei index and STI-derived deformation imaging in AL-CA patients

As mentioned before, the longitudinal function assessed by TDI derived strain was reduced in patients with AL amyloidosis, which also had an important prognostic relevance [22]. In the present study, apart from conventional echocardiographic indices, we focused on the comparison of prognosis performance between the Tei index and STI-derived deformation predictors. Deformation imaging has been well recognized over the last decade as a novel technology, which provides more accurate and sensitive assessment of global and segmental myocardial function in various cardiac conditions. The diagnostic and prognostic values of STI-derived strain rate measurements in CA patients have been demonstrated by our group and others [6, 22, 23]. Consistent with our previous study [7], GLSR_dias_ and E/GLS_dias_ independently predicted outcome in this cohort. However, the prognostic power of all these deformation markers was somehow weaker than that of the Tei index (c-statistics: GLS_sys_ 0.77, E/GLSR_dias_ 0.72, Tei index 0.85). Since good imaging quality is the essential prerequisite of the measurements of GLSR_dias_ and E/GLS_dias_, while Tei index could be reliably and easily obtained even in patients with unsatisfactory imaging condition, the present study underscores the priority of using Tei index instead of GLSR_dias_ and E/GLS_dias_ for the outcome assessment of AL-CA patients.

We further investigated the incremental predictive power of the Tei index and deformation imaging parameters. In particular, thresholds of Tei index ≥0.9 and GLS_sys_ ≤13% were analyzed (Fig. 3). We found that this combination performed well in predicting 1-year all-cause mortality with perfect specificity (100%) and positive predictive value (100%), negative predictive value of 80%, whereas sensitivity was 47%. Of note, the prediction of survival was also very good when this combination was used, with a specificity of 95% (but with a low sensitivity of 38%). Our results thus suggest that the combination of Tei index and GLS_sys_ can serve as a valuable tool to improve risk stratification in AL-CA patients. This might have important clinical implications regarding therapy regime, such as differentiating patients eligible for standard or high-dose chemotherapy, or to identify patients in need of stem cell replacement or heart transplantation.

Since NYHA functional class was a dominant predictor of mortality risk in this cohort, we further analyzed the prognostic role of Tei index in subgroups of NYHA class I–II vs class III–IV, respectively. The results showed that in the subgroup of NYHA class I–II, the Tei index was significantly higher in non-survivors than in survivors (0.81 ± 0.26 vs. 0.59 ± 0.13, p = 0.007). Likewise, in patients with NYHA class III–IV, the Tei index also tended to be higher in non-survivors than survivors, but this trend did not reach statistical significance (0.80 ± 0.31 vs. 0.64 ± 0.22, p = 0.152). These results suggest that the Tei index might be useful to predict prognosis of CA patients without overt clinical heart failure (NYHA class I–II), whereas the predictive value of the Tei index was somehow weaker in CA patients with more advanced heart failure symptoms (NYHA class III–IV). These findings need to be confirmed in future prospective studies with larger sample sizes.

### Technical considerations

In the present study, STI derived global strain was obtained from 4-chamber apical views only. In a pilot study, we compared the difference of global longitudinal strain value derived from all three apical views and a single 4-chamber apical view in 20 AL-CA patients, and observed similar strain values derived from all three apical views (GLS_sys_ −10.9 ± 3.7%) and a single 4-chamber apical view (GLS_sys_ −10.7 ± 4.4%, p = 0.674). Since imaging quality of the apical 4-chamber view is in usually superior compared to apical 2-chamber and long-axis views, the evaluation was thus performed based on apical 4-chamber view in the present study to shorten the evaluation time and pave the way for broader use of this technique in real world clinical situations.

### Study limitations

The current cohort included only a relatively small sample of a single subtype of cardiac amyloidosis, AL amyloidosis. The prognostic utility of the Tei index in combination with longitudinal strain should be validated in other subtypes such as hereditary or wild-type transthyretin amyloidosis. This might have particularly clinical relevant implications, as a specific medical therapy of transthyretin amyloidosis is expected to be clinically available very soon. Future clinical studies with ideally larger sample sizes are warranted to externally validate the proposed risk stratification strategy, compare the findings between the various entities of cardiac amyloidosis, and evaluate potential implications on therapy planning.

## Conclusions

Both Tei index and STI-derived deformation parameters can be used to predict mortality risk in AL-CA patients with superior prognostic value compared to conventional echocardiographic parameters. However, the prognostic power of deformation markers is somehow weaker than that of the Tei index. The Tei index can easily be obtained, even under difficult imaging conditons. The combination of Tei index and selected deformation parameters, such as Tei index plus GLS_sys_, provides excellent predictive utility for 1-year outcome and might therefore prove clinically valuable for risk stratification in this clinically demanding patient group.

## References

[1] Quarta CC, Kruger JL, Falk RH (2012). Cardiac amyloidosis. Circulation.

[2] Banypersad SM, Moon JC, Whelan C, Hawkins PN, Wechalekar AD (2012). Updates in cardiac amyloidosis: a review. J Am Heart Assoc.

[3] Cueto-Garcia L, Reeder GS, Kyle RA, Wood DL, Seward JB, Naessens J, Offord KP, Greipp PR, Edwards WD, Tajik AJ (1985). Echocardiographic findings in systemic amyloidosis: spectrum of cardiac involvement and relation to survival. J Am Coll Cardiol.

[4] Kristen AV, Perz JB, Schonland SO, Hansen A, Hegenbart U, Sack FU, Goldschmidt H, Katus HA, Dengler TJ (2007). Rapid progression of left ventricular wall thickness predicts mortality in cardiac light-chain amyloidosis. J Heart Lung Transplant.

[5] Patel AR, Dubrey SW, Mendes LA, Skinner M, Cupples A, Falk RH, Davidoff R (1997). Right ventricular dilation in primary amyloidosis: an independent predictor of survival. Am J Cardiol.

[6] Liu D, Hu K, Niemann M, Herrmann S, Cikes M, Stork S, Beer M, Gaudron PD, Morbach C, Knop S, Geissinger E, Ertl G, Bijnens B, Weidemann F (2013). Impact of regional left ventricular function on outcome for patients with AL amyloidosis. PLoS One.

[7] Liu D, Hu K, Stork S, Herrmann S, Kramer B, Cikes M, Gaudron PD, Knop S, Ertl G, Bijnens B, Weidemann F (2014). Predictive value of assessing diastolic strain rate on survival in cardiac amyloidosis patients with preserved ejection fraction. PLoS One.

[8] Buss SJ, Emami M, Mereles D, Korosoglou G, Kristen AV, Voss A, Schellberg D, Zugck C, Galuschky C, Giannitsis E, Hegenbart U, Ho AD, Katus HA, Schonland SO, Hardt SE (2012). Longitudinal left ventricular function for prediction of survival in systemic light-chain amyloidosis: incremental value compared with clinical and biochemical markers. J Am Coll Cardiol.

[9] Tei C, Dujardin KS, Hodge DO, Kyle RA, Tajik AJ, Seward JB (1996). Doppler index combining systolic and diastolic myocardial performance: clinical value in cardiac amyloidosis. J Am Coll Cardiol.

[10] Tei C, Ling LH, Hodge DO, Bailey KR, Oh JK, Rodeheffer RJ, Tajik AJ, Seward JB (1995). New index of combined systolic and diastolic myocardial performance: a simple and reproducible measure of cardiac function–a study in normals and dilated cardiomyopathy. J Cardiol.

[11] Schaefer A, Meyer GP, Hilfiker-Kleiner D, Brand B, Drexler H, Klein G (2005). Evaluation of Tissue Doppler Tei index for global left ventricular function in mice after myocardial infarction: comparison with Pulsed Doppler Tei index. Eur J Echocardiogr.

[12] Liu D, Hu K, Niemann M, Herrmann S, Cikes M, Stork S, Gaudron PD, Knop S, Ertl G, Bijnens B, Weidemann F (2013). Effect of combined systolic and diastolic functional parameter assessment for differentiation of cardiac amyloidosis from other causes of concentric left ventricular hypertrophy. Circ Cardiovasc Imaging.

[13] Nagueh SF, Appleton CP, Gillebert TC, Marino PN, Oh JK, Smiseth OA, Waggoner AD, Flachskampf FA, Pellikka PA, Evangelisa A (2009). Recommendations for the evaluation of left ventricular diastolic function by echocardiography. Eur J Echocardiogr.

[14] Hu K, Liu D, Herrmann S, Niemann M, Gaudron PD, Voelker W, Ertl G, Bijnens B, Weidemann F (2013). Clinical implication of mitral annular plane systolic excursion for patients with cardiovascular disease. Eur Heart J Cardiovasc Imaging.

[15] Marwick TH (2013). Methods used for the assessment of LV systolic function: common currency or tower of Babel?. Heart.

[16] Harjai KJ, Scott L, Vivekananthan K, Nunez E, Edupuganti R (2002). The Tei index: a new prognostic index for patients with symptomatic heart failure. J Am Soc Echocardiogr.

[17] Sasao H, Noda R, Hasegawa T, Endo A, Oimatsu H, Takada T (2004). Prognostic value of the Tei index combining systolic and diastolic myocardial performance in patients with acute myocardial infarction treated by successful primary angioplasty. Heart Vessels.

[18] Dujardin KS, Tei C, Yeo TC, Hodge DO, Rossi A, Seward JB (1998). Prognostic value of a Doppler index combining systolic and diastolic performance in idiopathic-dilated cardiomyopathy. Am J Cardiol.

[19] Blanchard DG, Malouf PJ, Gurudevan SV, Auger WR, Madani MM, Thistlethwaite P, Waltman TJ, Daniels LB, Raisinghani AB, DeMaria AN (2009). Utility of right ventricular Tei index in the noninvasive evaluation of chronic thromboembolic pulmonary hypertension before and after pulmonary thromboendarterectomy. JACC Cardiovasc Imaging.

[20] Migrino RQ, Mareedu RK, Eastwood D, Bowers M, Harmann L, Hari P (2009). Left ventricular ejection time on echocardiography predicts long-term mortality in light chain amyloidosis. J Am Soc Echocardiogr.

[21] Gibson DG, Francis DP (2003). Clinical assessment of left ventricular diastolic function. Heart.

[22] Koyama J, Falk RH (2010). Prognostic significance of strain Doppler imaging in light-chain amyloidosis. JACC Cardiovasc Imaging.

[23] Ternacle J, Bodez D, Guellich A, Audureau E, Rappeneau S, Lim P, Radu C, Guendouz S, Couetil JP, Benhaiem N, Hittinger L, Dubois-Rande JL, Plante-Bordeneuve V, Mohty D, Deux JF, Damy T (2016). Causes and Consequences of Longitudinal LV Dysfunction Assessed by 2D Strain Echocardiography in Cardiac Amyloidosis. JACC Cardiovasc Imaging.

